# Extreme Phenotypic Variability of *ACTG1*‐Related Disorders in Hearing Loss

**DOI:** 10.1002/ggn2.202400040

**Published:** 2024-12-05

**Authors:** Maria T. Bernardi, Memoona Ramzan, Laura Calderon, Franco Salvatore, Maria Agustina De Rosa, Stephanie Bivona, Romina Armando, Natalia Vazquez, Maria Esnaola Azcoiti, Marcelo A. Marti, Claudia Arberas, Maria Gabriela Ropelato, Silvina Olha, Byron L. Lam, Fred F. Telischi, Mustafa Tekin, Katherina Walz

**Affiliations:** ^1^ Instituto de Química Biológica de la Facultad de Ciencias Exactas y Naturales (IQUIBICEN) CONICET Buenos Aires 1428 Argentina; ^2^ John P. Hussman Institute for Human Genomics University of Miami Miller School of Medicine Miami FL 33136 USA; ^3^ Hospital de Niños Dr. R. Gutierrez Buenos Aires 1330 Argentina; ^4^ Dr. John T. Macdonald Foundation Department of Human Genetics University of Miami Miller School of Medicine Miami FL 33136 USA; ^5^ Sección Genética Médica Hospital de Niños Dr. R. Gutierrez Buenos Aires 1330 Argentina; ^6^ Centro de Investigaciones Endocrinológicas “Dr. César Bergadá” (CEDIE) CONICET – FEI – División de Endocrinología Hospital de Niños Ricardo Gutiérrez Buenos Aires 1330 Argentina; ^7^ Departamento de Química Biológica Facultad de Ciencias Exactas y Naturales Universidad de Buenos Aires (FCEyN‐UBA) Buenos Aires 1428 Argentina; ^8^ Bascom Palmer Eye Institute University of Miami Miller School of Medicine Miami FL 33136 USA; ^9^ Departments of Otolaryngology Neurological Surgery and Biomedical Engineering Miller School of Medicine University of Miami Miami FL 33136 USA

**Keywords:** ACTG1, hearing loss

## Abstract

Hearing loss is the most common sensory defect in humans, affecting normal communication. In most cases, hearing loss is a multifactorial disorder caused by both genetic and environmental factors, but single‐gene mutations can lead to syndromic or non‐syndromic hearing loss. Monoallelic variants in *ACTG1*, coding for gamma (γ)‐actin, are associated with classical Baraitser‐Winter Syndrome type 2 (BRWS2, nonsyndromic deafness, and a variety of clinical presentations not fitting the original BRWS2 description or nonsyndromic deafness. Here two unrelated patients with *ACTG1* variants are reported, having severe hearing loss as a common phenotype but with different clinical presentations, supporting the extreme variability of *ACTG1*‐related disorders.

## Graphical abstract

Different variants in a single gene can confer genetic pleiotropy (different phenotypes). One example is monoallelic variants in the *ACTG1*, coding for gamma (γ)‐actin, which is associated with classical Baraitser‐Winter Syndrome type 2 (BRWS2) and a variety of clinical presentations not fitting the original BRWS2 description including non‐syndromic deafness. Here two unrelated patients with *ACTG1* variants are reported, having severe hearing loss as a common phenotype but with different clinical presentations, supporting the extreme variability of *ACTG1*‐related disorders.

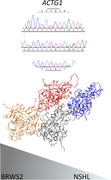

## 1 Introduction

Clinically significant hearing loss is present in at least 1.9 per 1000 infants at birth in the U.S.^[^ ^1^ ^]^ and the prevalence rises to at least 2.7 per 1000 by the age of four. At least 60% of congenital deafness is monogenic with a high degree of genetic heterogeneity. The presentation of monogenic forms of hearing loss can be syndromic (characterized by hearing loss in combination with other abnormalities) or nonsyndromic (with only hearing loss). Moreover, certain hearing loss‐related genes can cause both syndromic and nonsyndromic deafness. That is the case of *ACTG1* (MIM 102560) that causes BRWS2; (MIM 614583), a rare autosomal dominant (AD) condition with developmental delay/intellectual disability of variable degree and craniofacial dysmorphisms as the main clinical presentations. Brain abnormalities (especially pachygyria), microcephaly, epilepsy, as well as early onset progressive hearing loss, and cardiovascular and genitourinary abnormalities, may be present.^[^ ^2^, ^3^ ^]^ In addition, mutations in *ACTG1* cause autosomal dominant, non‐syndromic hereditary hearing loss DFNA20/26 (OMIM 604717), characterized by a post‐lingual‐onset and progressive sensorineural hearing loss (SNHL).^[^ ^4^ ^]^ It was suggested that BRWS2 represented the severe end of a spectrum of cytoplasmic actin‐associated phenotypes that extend to nonsyndromic hearing loss.^[^ ^5^ ^]^

Here we describe two patients carrying likely pathogenic variants in the *ACTG1* gene, with different clinical presentations but sensorineural hearing loss as one common feature. Our finding adds evidence to the extreme variability and broad spectrum of *ACTG1*‐related disorders, ranging from classical BRWS2 to nonsyndromic deafness, and occasionally new observed clinical features.

## 2 Discussion

The *ACTG1* gene, located on chromosome 17q25.3, encodes for gamma (γ)‐actin, a member of the actin family of globular proteins involved in a myriad of processes including embryonic structural and functional differentiation, cell motility, and maintenance of the cytoskeleton.^[^ ^6^ ^]^ There are at least six types of actin isoforms, each of them encoded by a separate gene, with very similar amino acid sequences,^[^ ^6^, ^7^, ^8^ ^]^ expressed at specific ratios in every cell type.^[^ ^8^ ^]^

γ‐actin (encoded by the *ACTG1 gene*, MIM# 102560), and β‐Actin (encoded by the *ACTB* gene, MIM# 102630) are conserved from birds to mammals, only differing by four biochemically similar residues.^[^ ^8^ ^]^ γ‐actin is abundant in the inner ear, specifically in hair cells and is specifically required for the maintenance and function of cytoskeletal structures in the ear.^[^ ^9^ ^]^ Several HL‐related pathogenic variants have been reported in ACTG1 without clustering in a specific hot spot but rather distributed throughout the actin monomer.^[^ ^10^ ^]^

Dominantly inherited mutations in *ACTG1* have been described in BRWS2, characterized by variable degrees of developmental delay/intellectual disability and distinct craniofacial findings as the main clinical presentations. Additionally, brain abnormalities (especially pachygyria), microcephaly, epilepsy, as well as early onset progressive hearing loss, cardiovascular and genitourinary abnormalities may be present.^[^ ^2^, ^3^, ^5^, ^11^, ^12^ ^]^ Widely spaced eyes, bilateral ptosis, and highly arched eyebrows are present in 90% or more of the patients while abnormal ears and trigonocephaly are present in over 60% of the patients. Developmental delay and intellectual disability are observed in 95% of the patients and are related to the severity of the brain malformations. Epilepsy and vision abnormalities are often reported in these individuals.

We compared the clinical features of Patients 1 and 2 with the phenotypic spectrum of BRWS2, summarized in **Table** 1. While similarities were found with the typical features of the syndrome, certain characteristics of the individuals do not fully align. Patient 1 presents certain dysmorphic craniofacial features, which are consistent with the majority of individuals with this syndrome. He also exhibits bilateral ptosis and abnormal ears. These are not present in Patient 2. Both patients showed developmental delay and intellectual disability, and early onset of severe sensorineural hearing loss. In Patient 2, the presence of brain malformations was evidenced, precisely a left parietal lobe developmental venous anomaly. This information is not available for Patient 1. There is no data on the existence of epilepsy episodes in either of the two cases. While one typical feature of this syndrome is microcephaly, Patient 1 presents with macrocephaly, an intriguing discrepancy in this case. On the other hand, no information was found regarding this data for Patient 2. Further, vision abnormalities were also reported in both patients; Patient 1 was diagnosed with myopia, astigmatism, and evasive visual behavior, while Patient 2 had retinitis pigmentosa. It is important to note that *ACTG1* c.*773C>T* (p.Pro258Leu) was previously reported in a patient with a similar clinical presentation to Patient 2: progressive sensorineural hearing loss, intellectual disability, and interestingly enough, visual impairments, an uncommon feature in BWS2.^[^ ^13^ ^]^

**Table 1:** Clinical features of Patient 1 and Patient 2 compared with the phenotypic spectrum of the BRW2S.

		Patient 1	Patient 2
Gender		Male	Female
Variant		*ACTG1* c.494T>C (p.Ile165Thr)	*ACTG1* c.773C>T (p.Pro258Leu)

BRWS2 core features	Frequency^a)^		
Dysmorphic craniofacial	100%	Yes	NA
Developmental delay/intellectual disability	>95%	Yes	Yes
Presence of brain malformations	60–70%	NA	Yes
Epilepsy	50%	NA	NA
Microcephaly	≈50%	No	NA
Hearing loss/deafness	35%	Yes	Yes
Vision abnormality	30%	Yes	Yes

^a)^ https://www.ncbi.nlm.nih.gov/books/NBK327153/.^[^ ^11^ ^]^

On the other hand, we compare our patients with most cases of DFNA20/26 hearing loss where hearing loss begins in the first to third decades of life, and progresses to profound deafness by the sixth decade of life. The patients presented here have prelingual onset, and severe hearing loss not fitting the canonical DFNA20/26 presentation.

In summary, here we described two patients carrying likely pathogenic variants in the *ACTG1* gene, with different clinical presentations but sensorineural hearing loss as one common feature, reinforcing the extreme variability of *ACTG1*‐related disorders.

## 3 Experimental Section

### Ethics Statement and Study Participants

Families were enrolled as part of a larger cohort for the identification of novel genes involved in sensorineural HL. The study was approved by the Institutional Review Board at the University of Miami, USA, and the Hospital de Niños Dr. R. Gutierrez, Ethics Committee, Argentina; according to the “World Medical Association Declaration of Helsinki.” Written informed consents were obtained from all participants and in the case of minors from parents. Audiological examinations were tested as per standard procedures and guidelines.^[^ ^14^ ^]^ Medical history and biochemical tests were requested where required.

### Genetic Analysis

Genomic DNA from the probands and parents was extracted from peripheral venous blood cells using Gentra Puregene Blood Kit (Qiagen). DNA samples from both probands were screened and found negative for the presence of biallelic variants in *GJB2* (MIM 121011) and deletions of *GJB6* (MIM 604418), followed by Exome Sequencing. Libraries were performed with an in‐house PCR‐free library preparation protocol at BGI Genomics. Sequencing of the samples was carried out using a BGISEQ‐500 with paired‐end 100 bp (PE100). The data obtained was aligned against human GRCh37/hg19 genome assembly using Burrows–Wheeler Aligner (http://bio‐bwa.sourceforge.net) and variant calling was done with the GATK software package (https://www.broadinstitute.org/gatk/). The FASTQ files were analyzed with an in‐house software GENESIS (https://app.tgp‐foundation.org). All exonic sequences, including splice sites, were analyzed for the presence of frameshift, missense, and nonsense variants in a custom‐defined list of known deafness genes (Table S1, Supporting Information). Variants were retained for further evaluation if they had an allele frequency of less than 0.05, except for genes with known high‐frequency variants (i.e., connexins) that were analyzed individually. Homozygous, hemizygous, and compound heterozygous variants were examined. Confirmation and Segregation studies by Sanger sequencing were performed when appropriate. Copy number variants (CNVs) analysis with ES data used CoNIFER v.02.2 with default parameters. It used a singular value decomposition method to correct systematic biases and identified a CNV call if the corrected signal reached a predefined threshold at no less than three consecutive exons.^[^ ^15^ ^]^ The pathogenicity of the detected variants was determined as per the description by the ACMG and ClinGen Hearing Loss Expert Panel specifications to the ACMG/AMP variant interpretation guidelines^[^ ^16^, ^17^ ^]^ and AlphaMissense.^[^ ^18^ ^]^ A summary of the variant characteristics is presented in Table S2 (Supporting Information).

### Structural Analysis

The structural context was analyzed using the Cryo‐EM structure of nonmuscle gamma‐actin (PDBid 8DNF), which contained an actin tetramer. For each variant, the following structural‐thermodynamic parameters were computed, FoldX change in folding free energy,^[^ ^19^ ^]^ utilizing the “PossitionScan” command for calculations that return the stability ΔΔG energy score of a given mutation. Changes in hydropathy and conservation index were tested using BLOSUM62 matrix,^[^ ^20^ ^]^ secondary and tertiary (buried, interface, or surface) environment, and visual inspection of residue‐residue interactions with DDMut.^[^ ^21^ ^]^

### Clinical Report—Patient 1

The first patient was a 9‐year‐old male, who was seen by the Otorhinolaryngologic Service at the Hospital de Niños Dr.R. Gutierrez in Argentina, due to hearing loss. The patient was diagnosed with prelingual‐onset bilateral severe to profound SNHL by brainsteam evoked response audiometry (BERA) performed at age 3 years due to lack of language. He was the second child born to a non‐consanguineous 30‐year‐old mother and a 33‐year‐old father. Family history included a miscarriage but was otherwise unremarkable. Prenatal history was not complicated, except for a urinary infection at 7 months of gestation treated with antibiotics. No fetal anomalies were documented by first and second‐trimester prenatal ultrasound (US) imaging. He was delivered by C‐section due to a nuchal cord. Unremarkable audiologic and metabolic screening tests at birth.

At 3 years of age, the patient was non‐verbal, only monosyllabic words, relying on gestures to understand others. No psychomotor developmental delay was noticed. He sat up at 6 months of age, walked independently at 13 months, and had sphincter control before 3 years of age (all normal range). Normal temporal bone by CT scan without contrast. Cochlear implantation was performed at the age of 4 years and speech therapy was initiated. He attended a special needs school due to reading and learning delays. At the time of the last evaluation at age 9 years, he presented with obesity, weight was 58 kg (+7 SD); height was 144 cm (97th percentile), and head circumference was 58 cm (+4 SD, macrocephaly). Craniofacial characteristics such as round skull and face, flat forehead, sunken eyes, bilateral epicanthal fold, round nose tip, fleshy nostrils, short and wide philtrum, fleshy mouth, high palate, open bite, small chin, and fleshy ears were noted. Other observed characteristics were lordosis, prominent abdomen, and dry and thick skin with topical eczema. During walking, lower limb genu valgum and flat valgus feet were observed. In addition, myopia, astigmatism, as well as evasive visual behavior without retinal abnormalities were detected. By exome sequencing an *ACTG1*, c.494T>C (p.Ile165Thr) *de novo* variant was found (Table S2, Supporting Information). No other single nucleotide, indel, or copy number variant in a recognized deafness gene were detected.

### Clinical Report—Patient 2

The second patient was a 33‐year‐old white female, who was evaluated at the University of Miami Hearing Loss Clinic. Medical history and audiological evaluations showed congenital or prelingual‐onset, progressive bilateral SNHL that was first noted with a lack of response to loud sounds at age 18 months. Her hearing loss was bilateral and profound at age 27. She was the second child of non‐consanguineous parents with an unremarkable family history.

Medical history included global developmental delay during infancy and mild intellectual disability. MRI of the brain showed mild white matter changes without malformations in the ear or brain. She had a unilateral cochlear implant at age 30. Around age 2 exotropia of the left eye was noticed and strabismic surgery at age 3 was performed. She had patching and glasses as a child. She started complaining of blurred vision, night vision problems, and nyctalopia at age 27. Retinal examination suggested retinal degeneration which was confirmed by electroretinography. On physical exam, her head circumference was 54 cm; she had a strabismus of the left eye, bulbous nasal tip, and short appearing philtrum. Exome sequencing revealed that the patient was heterozygous for *ACTG1*, c.*773C>T* (p.Pro258Leu), which was likely pathogenic (Table S2, Supporting Information). Parents were not available for testing.

### In Silico Analysis of Variants Pathogenicity

High conservation was well known for the actin protein family.^[^ ^22^ ^]^ In concordance, *ACTG1* showed a low tolerance to missense variations, as reflected by a GnomAD Z‐score of 4.88.^[^ ^23^ ^]^ Ile165 and Pro258 were highly conserved from humans to zebrafish in ACTG1 (Figure S1, Supporting Information), and fully conserved between all actin isoforms.^[^ ^24^ ^]^ AlphaMissense^[^ ^18^ ^]^ predicted a pathogenic outcome for Ile165Thr and Pro258Leu missense variations. Interestingly, for both residues and their neighboring residues, 8 and 5 respectively, almost all possible missense variations were predicted as pathogenic. Out of 247 possible variants, 235 of them were described as pathogenic (95 %), reinforcing their structural importance.

**Figure 1:**
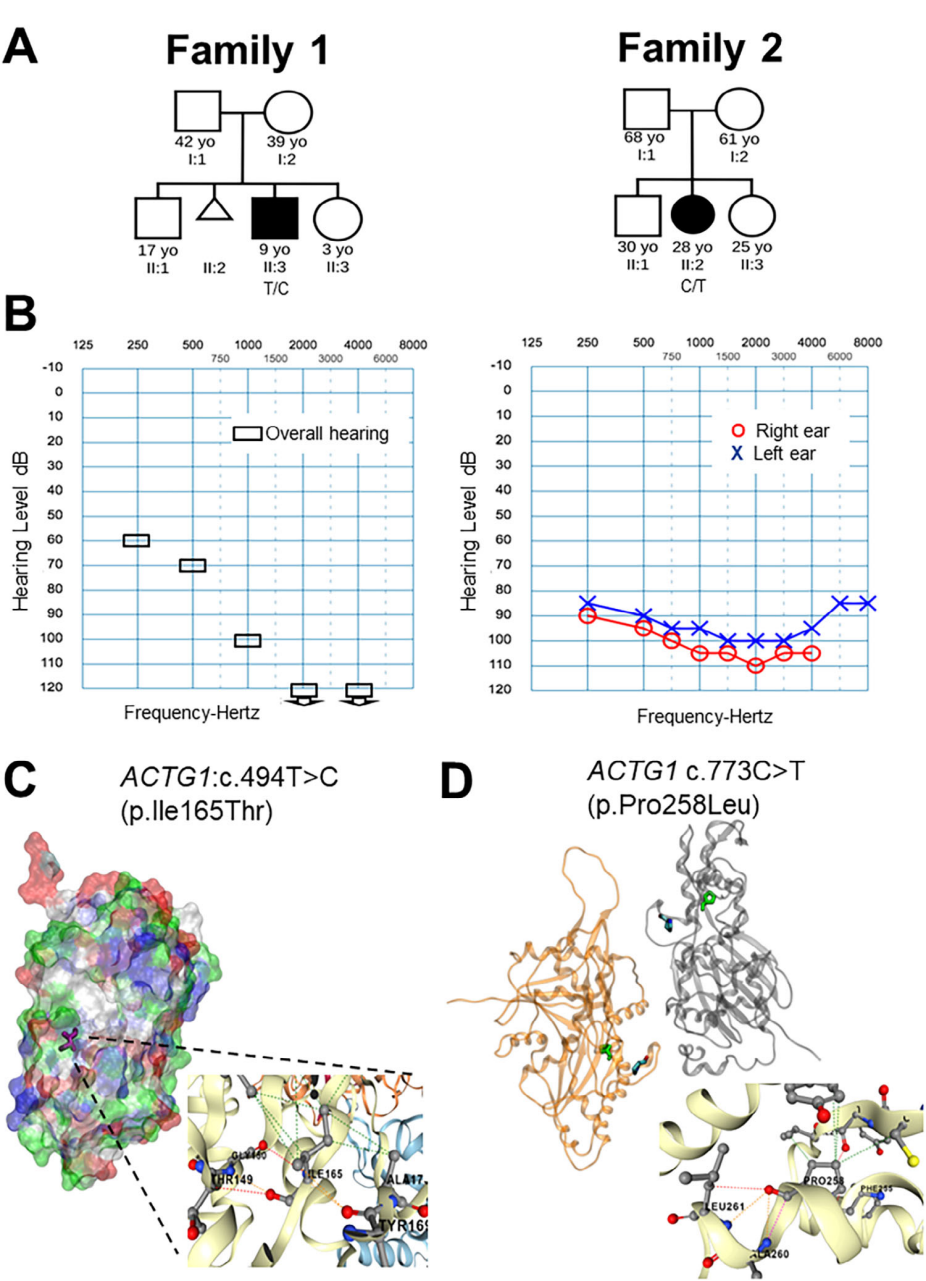
Clinical and molecular evaluation of hearing loss patients A) Pedigrees of affected individuals (marked with arrows). Age is shown for the patients on their first visit. B) Tone audiometry, Suzuki–Ogiba test^[^ ^25^ ^]^ Black rectangles: Overall hearing, for Patient 1 and Pure‐tone audiometry (Red circles: Right ear; Blue crosses: Left ear) for Patient 2 are depicted. Both audiograms are faithful copies of the original studies. C) In silico modeling of the variants. 3D Structural visualization of γ‐actin. The image shows the monomer chain B, with the Ile165 in magenta, and molecular surface representation colored by chemical classification of residues; hydrophobic (white), hydrophilic (green), positive charged (blue), and negative charged (red). D) Dimer Chain C and D, Pro 258 (green) and Pro264 (blue). These two prolines are responsible for the loop that presumably stabilizes the dimer by interacting with the neighbor chain. In both cases, zoom‐in shows the reference residue and the type of interactions with their neighbor residues; polar (orange), hydrophobic (green), hydrogen bond (red), and clash (pink).

Actin polypeptides were found as monomers (G‐form) and filaments (F‐form) and both structures had been solved. Visual inspection of both variants showed that Ile165 was located close to the actin‐actin interaction interphase, at the end of a beta‐sheet, forming part of a hydrophobic cluster together with Ala170, Leu171, and Leu293, as well as Met43 from the neighboring chain, which resulted in its being completely buried (**Figures** **1**C, S1B, Supporting Information). Substitution for hydrophilic Threonine was expected to disrupt this hydrophobic cluster, as consistently predicted by FoldX and Blosum 62 Classification (Table S3, Supporting Information).

On the other hand, Pro258 was in a 3‐10‐helix of the actin fold core. The change to Leucine might conserve hydrophobic interactions, explaining why the FoldX Stability Classification for the variant was “Benign.” In contrast, the delta of Blosum62 for Pro258Leu was extremely low, meaning that the mutation tended to be very disruptive in most proteins (Table S3, Supporting Information). The presence of Pro258 and Pro263 could be key to breaking and turning the helix of the longitudinal inter‐protomer interface, which binds to the D‐loop, required for proper actin polymerization^[^ ^24^ ^]^ hence, Pro258Leu might disrupt the polymerization. The polymer structure showed how Pro258 and Pro263 formed a loop that interacts with the neighbor chain (Figure 1D). A mutation that disrupts loop structure, such as Pro258Leu, could impair proper actin polymerization (Figure S1, Supporting Information) leading to a loss of normal function and disease.

### Ethics Declaration

The study was approved by the Institutional Review Board at the University of Miami, USA, the Ankara University Medical School Ethics Committee, Turkey, and the Hospital de Niños Dr. R. Gutierrez, Ethics Committee, Argentina; according to the “World Medical Association Declaration of Helsinki”.

## Conflict of interest

The authors declare no conflict of interest.

## Author Contributions

K.W., M.T. conceptualized the study. K.W., and M.T. collected data. M.T.B., M.R., L.C., F.S., M.A.D.R., S.B., R.A., N.V., R.R., M.A.M., C.A., G.R., S.O., B.L.L., F.F.T., M.T., and K.W. performed formal analysis. K.W. and M.T. acquired funds. M.T.B., K.W., A.d.R., and M.R. wrote the original manuscript. Writing‐review and editing: M.T.B., M.R., L.C., F.S., M.A.D.R., S.B., R.A., N.V., R.R., M.A.M., C.A., G.R., S.O., B.L.L., F.F.T., M.T., and K.W. wrote, reviewed, and edited the final manuscript.

## Supporting information



Supporting Information

## Data Availability

The data that support the findings of this study are available in the supplementary material of this article.

## References

[1] C. C. Morton , W. E. Nance , N. Engl. J. Med. 2006, 354, 2151. 16707752 10.1056/NEJMra050700

[2] O. F. Chacon‐Camacho , T. Barragan‐Arevalo , C. E. Villarroel , M. Almanza‐Monterrubio , J. C. Zenteno , Eur. J. Med. Genet. 2020, 63, 103877. 32028042 10.1016/j.ejmg.2020.103877

[3] T. M. Yates , C. L. Turner , H. V. Firth , J. Berg , D. T. Pilz , Clin. Genet. 2017, 92, 3. 27625340 10.1111/cge.12864

[4] M. Zhu , T. Yang , S. Wei , A. T. DeWan , R. J. Morell , J. L. Elfenbein , R. A. Fisher , S. M. Leal , R. J. H. Smith , K. H. Friderici , Am. J. Hum. Genet. 2003, 73, 1082. 13680526 10.1086/379286 PMC1180488

[5] J. Riviere , B. W. M. van Bon , A. Hoischen , S. S. Kholmanskikh , B. J. O'Roak , C. Gilissen , S. Gijsen , C. T. Sullivan , S. L. Christian , O. A. Abdul‐Rahman , J. F. Atkin , N. Chassaing , V. Drouin‐Garraud , A. E. Fry , J. Fryns , K. W. Gripp , M. Kempers , T. Kleefstra , G. M. S. Mancini , M. J. M. Nowaczyk , C. M. A. van Ravenswaaij‐Arts , T. Roscioli , M. Marble , J. A. Rosenfeld , V. M. Siu , B. B. A. de Vries , J. Shendure , A. Verloes , J. A. Veltman , H. G. Brunner , et al., Nat. Genet. 2012, 44, 440. 22366783 10.1038/ng.1091 PMC3677859

[6] A. S. Kashina , Semin. Cell Dev. Biol. 2020, 102, 113. 32001148 10.1016/j.semcdb.2019.12.003 PMC7214208

[7] J. Vandekerckhove , K. Weber , J. Mol. Biol. 1978, 126, 783. 745245 10.1016/0022-2836(78)90020-7

[8] A. Simiczyjew , K. Pietraszek‐Gremplewicz , A. J. Mazur , D. Nowak , Histol. Histopathol. 2017, 32, 1125. 28439872 10.14670/HH-11-896

[9] J. Park , J. E. Bird , Hear. Res. 2023, 436, 108817. 37300948 10.1016/j.heares.2023.108817 PMC10408727

[10] U. Sorrentino , C. Piccolo , C. Rigon , V. Brasson , E. Trevisson , F. Boaretto , A. Martini , M. Cassina , Audiol. Res. 2021, 11, 582. 34698053 10.3390/audiolres11040052 PMC8544197

[11] A. Verloes , N Di Donato , J. Masliah‐Planchon , M. Jongmans , O. A. Abdul‐Raman , B. Albrecht , J. Allanson , H. Brunner , D. Bertola , N. Chassaing , A. David , K. Devriendt , P. Eftekhari , V. Drouin‐Garraud , F. Faravelli , L. Faivre , F. Giuliano , L Guion Almeida , J. Juncos , M. Kempers , H. K. Eker , D. Lacombe , A. Lin , G. Mancini , D. Melis , C. M. Lourenco , V. M. Siu , G. Morin , M. Nezarati , M. J. M. Nowaczyk , et al., Eur. J. Hum. Genet. 2015, 23, 292. 25052316 10.1038/ejhg.2014.95 PMC4326722

[12] S. Ghiselli , G. Parmeggiani , G. Zambonini , D. Cuda , J Clin. Med. 2024, 13, 1500. 38592426 10.3390/jcm13051500 PMC10935159

[13] C. Zazo Seco , M. Wesdorp , I. Feenstra , R. Pfundt , J. Y. Hehir‐Kwa , S. H. Lelieveld , S. Castelein , C. Gilissen , I. J. de Wijs , R. J. Admiraal , R. J. Pennings , H. P. Kunst , J. M. van de Kamp , S. Tamminga , A. C. Houweling , A. S. Plomp , S. M. Maas , P. A. de Koning Gans , S. G. Kant , C. M. de Geus , S. G. Frints , E. K. Vanhoutte , M. F. van Dooren , M. H. van den Boogaard , H. Scheffer , M. Nelen , H. Kremer , L. Hoefsloot , M. Schraders , H. G. Yntema , Eur. J. Hum. Genet. 2017, 25, 308. 28000701 10.1038/ejhg.2016.182 PMC5315517

[14] M. Mazzoli , G. Van Camp , V. Newton , N. Giarbini , F. Declau , A. Parving , Audiol. Med. 2003, 1, 148.

[15] S. A. Gregoriades , Cah. Anesthesiol. 1990, 38, 347. 2285873

[16] M. T. DiStefano , S. E. Hemphill , A. M. Oza , R. K. Siegert , A. R. Grant , M. Y. Hughes , B. J. Cushman , H. Azaiez , K. T. Booth , A. Chapin , H. Duzkale , T. Matsunaga , J. Shen , W. Zhang , M. Kenna , L. A. Schimmenti , M. Tekin , H. L. Rehm , A. N. A. Tayoun , S. S. Amr , Genet. Med. 2019, 21, 2239. 30894701 10.1038/s41436-019-0487-0 PMC7280024

[17] S. Richards , N. Aziz , S. Bale , D. Bick , S. Das , J. Gastier‐Foster , W. W. Grody , M. Hegde , E. Lyon , E. Spector , K. Voelkerding , H. L. Rehm , Genet. Med. 2015, 17, 405. 25741868 10.1038/gim.2015.30 PMC4544753

[18] J. Cheng , G. Novati , J. Pan , C. Bycroft , A. Zemgulyte , T. Applebaum , A. Pritzel , L. H. Wong , M. Zielinski , T. Sargeant , R. G. Schneider , A. W. Senior , J. Jumper , D. Hassabis , P. Kohli , Z. Avsec , Sci. 2023, 381, eadg7492. 10.1126/science.adg7492 37733863

[19] J. Schymkowitz , J. Borg , F. Stricher , R. Nys , F. Rousseau , L. Serrano , Nucleic Acids Res. 2005, 33, W382. 15980494 10.1093/nar/gki387 PMC1160148

[20] S. Henikoff , J. G. Henikoff , Proc. Natl. Acad. Sci. USA 1992, 89, 10915. 1438297 10.1073/pnas.89.22.10915 PMC50453

[21] Y. Zhou , Q. Pan , D. E. V. Pires , C. H. M. Rodrigues , D. B. Ascher , Nucleic. Acids. Res. 2023, 51, W122. 37283042 10.1093/nar/gkad472 PMC10320186

[22] L. D. Bertola , E. B. Ott , S. Griepsma , F. J. Vonk , C. P. Bagowski , BMC Evol. Biol. 2008, 8, 166. 18518953 10.1186/1471-2148-8-166 PMC2443135

[23] K. E. Samocha , E. B. Robinson , S. J. Sanders , C. Stevens , A. Sabo , L. M. McGrath , J. A. Kosmicki , K. Rehnstrom , S. Mallick , A. Kirby , D. P. Wall , D. G. MacArthur , S. B. Gabriel , M. DePristo , S. M. Purcell , A. Palotie , E. Boerwinkle , J. D. Buxbaum , E. H. J. Cook , R. A. Gibbs , G. D. Schellenberg , J. S. Sutcliffe , B. Devlin , K. Roeder , B. M. Neale , M. J. Daly , Nat. Genet. 2014, 46, 944. 25086666 10.1038/ng.3050 PMC4222185

[24] A. S. Arora , H. Huang , R. Singh , Y. Narui , A. Suchenko , T. Hatano , S. M. Heissler , M. K. Balasubramanian , K. Chinthalapudi , eLife. 2023, 12, 82015. 10.7554/eLife.82015 PMC10072879 36790143

[25] T. Suzuki , Y. Ogiba , Arch. Otolaryngol. 1961, 74, 192.

