# Supplementary material for: Extreme Phenotypic Variability of ACTG1‐Related Disorders in Hearing Loss
Source: Adv Genet (Hoboken). 2024 Dec 5;5(4):2400040. doi: 10.1002/ggn2.202400040 (PMC11672310; doi:10.1002/ggn2.202400040)
Supplement: Supplementary file 1 — Supporting Information [file GGN2-5-2400040-s001.docx]

| HEARING LOSS GENES | |
| --- | --- |
| 121 Genes from Hereditary Hearing Loss Homepage^1^ | *ACTG1, ADCY1, AIFM1, BDP1, BSND, CABP2, CCDC50, CD164, CDC14A, CDH23, CEACAM16, CIB2, CLDN14, CLDN9, CLIC5, CLRN2, COCH, COL11A1, COL11A2, COL4A6, CRYM, DCDC2, DIABLO, DIAPH1, DMXL2, ELMOD3, EPS8, EPS8L2, ESPN, ESRP1, ESRRB, EYA4, FAM65B, GAB1, GIPC3, GJB2, GJB3, GJB6, GPSM2, GRAP, GRHL2, GRXCR1, GRXCR2, GSDME, HGF, HOMER2, IFNLR1, ILDR1, KARS, KCNQ4, KITLG, LHFPL5, LMX1A, LOXHD1, LRTOMT, MAP1B, MARVELD2, MCM2, MET, MINAR2, MPZL2, MSRB3, MYH14, MYH9, MYO15A, MYO3A, MYO6, MYO7A, NARS2, NLRP3, OSBPL2, OTOA, OTOF, OTOG, OTOGL, P2RX2, PCDH15, PDE1C, PDZD7, PJVK, PLS1, PNPT1, POU3F4, POU4F3, PPIP5K2, PRPS1, PTPRQ, RDX, REST, RIPOR2, ROR1, S1PR2, SCD5, SERPINB6, SIX1, SLC12A2, SLC17A8, SLC22A4, SLC26A4, SLC26A5, SMPX, SPNS2, STRC, SYNE4, TBC1D24, TECTA, TJP2, TMC1, TMEM132E, TMIE, TMPRSS3, TNC, TPRN, TRIOBP, TRRAP, TSPEAR, USH1C, USH2A, WBP2, WFS1, WHRN* |
| 163 Genes from Literature, commercial panels, and phenotype.^2-4^ | *A2ML1, ABHD12, ABHD5, ACOX1, ACTB, ADGRV1, ALMS1, ANLN, AP1B1, ARSB, ARSG, ASIC5, ATOH1, ATP1A3, ATP2B2, ATP6V1B1, ATP6V1B2, BCAP31, BCS1L, BTD, CACNA1D, CATSPER2, CD151, CEP250, CEP78, CHD7, CISD2, CLPP, CLRN1, COL1A1, COL2A1, COL4A3, COL4A4, COL4A5, COL9A1, COL9A2, COL9A3, DBH, DCAF17, DIAPH3, DNAJC3, DNMT1, DSPP, DTNA, EDN3, EDNRA, EDNRB, EFTUD2, ERAL1, EYA1, FDXR, FGF3, FGFR3, FITM2, FOXC1, FOXI1, GALNS, GATA3, GDF6, GJA1, GJB1, GLB1, GNS, GPRASP2, GREB1L, GUSB, HARS1, HARS2, HGSNAT, HOXA2, HSD17B4, HYAL1, IDS, IDUA, JAG1, KARS1, KCNE1, KCNJ10, KCNQ1, LARS2, LHX3, LOXL3, LRP2, MAFB, MAN2B1, MEOX1, MEPE, MIR96, MITF, MPZ, MRPS2, MYH7B, MYO18B, NAGLU, NDP, NDRG1, NF2, NOG, OPA1, PAX3, PCGF2, PEX1, PEX10, PEX11B, PEX12, PEX13, PEX14, PEX16, PEX19, PEX2, PEX26, PEX3, PEX5, PEX6, PEX7, PHYH, PLS3, PMP22, POLR1C, POLR1D, PRKCB, RAI1, REEP6, RMND1, RPS6KA3, SCP2, SEMA3E, SERAC1, SGSH, SH3TC2, SIX2, SIX5, SLC29A3, SLC44A4, SLC4A11, SLC52A2, SLC52A3, SLC9A1, SLITRK6, SNAI2, SOX10, TBX1, TCOF1, TFAP2A, TIMM8A, TMEM126A, TMEM43, TRMT10C, TSHZ1, TUBB4B, TWNK, UBR1, USH1G, VCAN, XYLT2, ZNF469, ANKH, CDKN1C, DFNA5, FGFR2, KMT2D, TYR, TOGARAM2* |

**Table S1.** List of analyzed genes. **^1^** <https://hereditaryhearingloss.org/> ^2^ https://www.ncbi.nlm.nih.gov/gtr/tests/567331/, ^3^ https://www.ncbi.nlm.nih.gov/gtr/tests/593052/, ^4^ https://www.ncbi.nlm.nih.gov/gtr/tests/553458/

|  | ***ACTG1* NM_001614.5** | |
| --- | --- | --- |
| Patient | 1 | 2 |
| Genomic position | chr17:g.81511496A>G | chr17:g.81511217G>A |
| Coding | c.494T>C | c.773C>T |
| Protein | p.Ile165Thr | p.Pro258Leu |
| Zygosity | Heterozygous | Heterozygous |
| Mutation type | Missense | Missense |
| ID | CA401460958 - rs2143778877 | CA295068193 - rs11549191 |
| Frequency  (GnomAD v3.1.2) | Absent | Absent |
| Revel Score | 0.965 | 0.895 |
| PhastCons100way | 1 | 1 |
| CADD (GRCh38-v1.7) | 32 | 28.9 |
| MAVERICK | AD 0.88 | N/A |
| Previously published  ClinVar variation ID | N/A  1683910 | PMID28000701  639093 |
| Domain | ACTIN domain | ACTIN domain |
| Classification | Likely Pathogenic (PP2+PP3_Moderate + PM6_Moderate + PM2_Supporting) | Likely Pathogenic (PS1_Strong +PP2+PP3_Moderate + PM2_Supporting) |

**Table S2.** DNA variants characteristics. The genomic positions are described in the GRCh38 version. N/A: not available. Classification according to Sequence Variant Interpretation Work Group recommendations and ACMG/AMP guidelines.

**
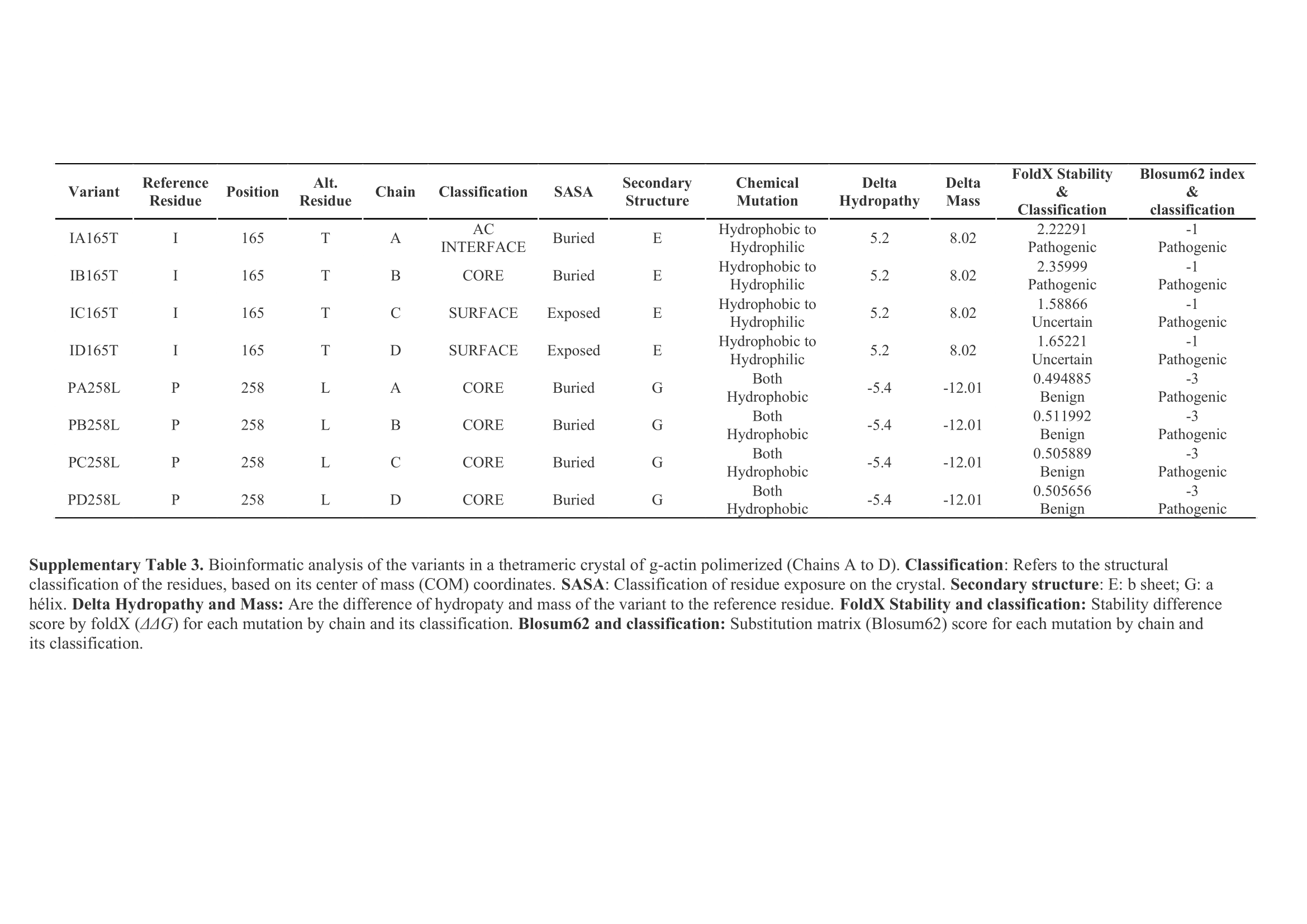
**

**Table S3.** Bioinformatic analysis of the variants in a tetrameric crystal of g-actin polymerized (Chains A to D). Classification: Refers to the structural classification of the residues, based on its center of mass (COM) coordinates. SASA: Classification of residue exposure on the crystal. Secondary structure: E: b sheet; G: a hélix. Delta Hydropathy and Mass: These are the differences between hydropathy and mass of the variant to the reference residue. FoldX Stability and classification: Stability difference score by foldX (*ΔΔG*) for each mutation by chain and its classification. Blosum62 and classification: Substitution matrix (Blosum62) score for each mutation by chain and its classification.


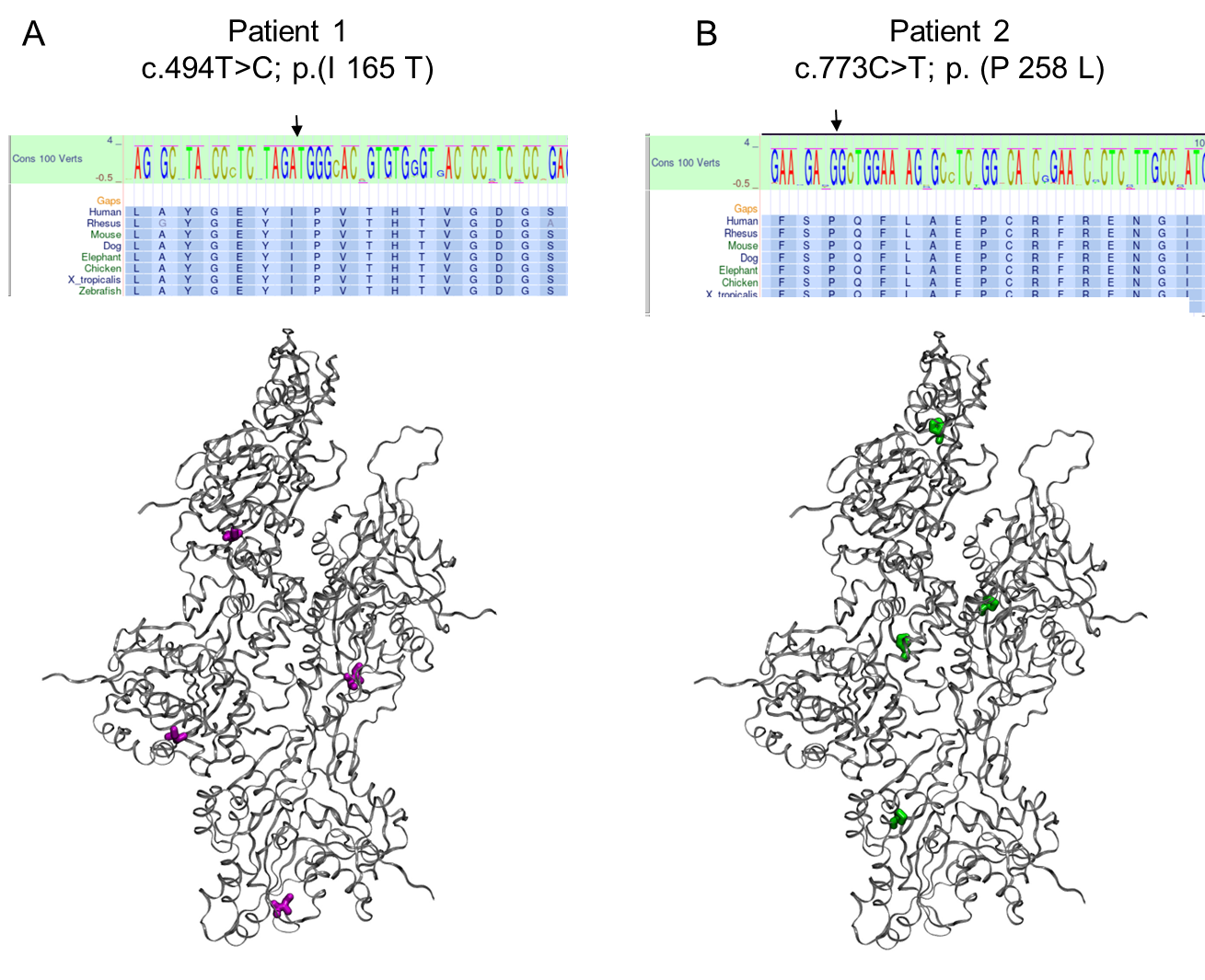


**Figure S1**: Conservation and location in the 3D Structural visualization of tetrameric γ-actin polymer for both mutated residues.
